# Vibrational Modes and Terahertz Phenomena of the Large-Cage Zeolitic Imidazolate Framework-71

**DOI:** 10.1021/acs.jpclett.2c00081

**Published:** 2022-03-24

**Authors:** Annika F. Möslein, Jin-Chong Tan

**Affiliations:** Multifunctional Materials and Composites (MMC) Laboratory, Department of Engineering Science, University of Oxford, Parks Road, Oxford OX1 3PJ, U.K.

## Abstract

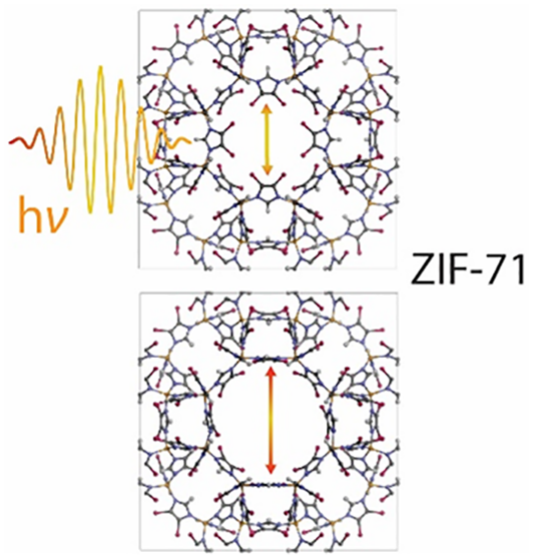

The zeolitic imidazole framework ZIF-71 has the potential to outperform other well-studied metal–organic frameworks due to its intrinsic hydrophobicity and relatively large pore size. However, a detailed description of its complex physical phenomena and structural dynamics has been lacking thus far. Herein, we report the complete assignment of the vibrational modes of ZIF-71 using high-resolution inelastic neutron scattering measurements and synchrotron radiation infrared spectroscopy, corroborated by density functional theory (DFT) calculations. With its 816 atoms per unit cell, ZIF-71 is the largest system yet for which frequency calculations have been accomplished employing the CRYSTAL17 DFT code. We discover low-energy terahertz dynamics such as gate-opening and shearing modes that are central to the functions and stability of the ZIF-71 framework structure. Nanoscale analytical methods based on atomic force microscopy (near-field infrared spectroscopy and AFM nanoindentation) further unravel the local chemical and mechanical properties of ZIF-71 single crystals.

Among the vast field of nanomaterials, metal–organic frameworks (MOFs) have gained considerable interest owing to their unique physical and chemical properties, which are unattainable in other conventional materials. For instance, their open framework structure assembled from metal clusters bridged by organic linkers leads to large surface areas even exceeding those of zeolites, while their organic–inorganic character offers novel, tailorable functional properties.^1^ Originating from the traditional use of porous nanomaterials, where MOFs have been proven beneficial for gas capture and storage, the multifunctional nature of MOFs has paved the way for an array of innovative applications, including but not limited to catalysis, drug delivery, microelectronics, and chemical sensors.^2−7^

One of the most promising candidates for the application of MOFs is the zeolitic imidazole framework ZIF-8 [Zn(mIM)_2_; mIM = 2-methylimidazolate] due to its stability and ease of synthesis.^8^ ZIF-8 crystallizes in a sodalite (SOD) topology with an internal pore size of ∼10 Å, and it has become a prototypical and well-studied material among the large family of MOFs.^9^ ZIF-8, or materials in the subclass of ZIFs, in general, are constructed from metal cations tetrahedrally coordinated to imidazole-type organic linkers, yielding a chemically stable framework structure with cage-like subunits.^8^ While ZIF-8 has indeed sparked considerable scientific and technological interests, other ZIF materials, in fact, might even outperform ZIF-8 in various applications.^10,11^ For instance, the far less studied material ZIF-71, built from Zn cations bridged by 4,5-dichloroimidazolate (dcIM) linkers, possesses a RHO-type structure with pore sizes exceeding those of the SOD-type ZIF-8, thus rendering ZIF-71 a promising candidate for enhanced gas capture or mechanical shock absorbance.^12^ It crystallizes in a cubic symmetry, and it is constructed from large α-cages (16.5–16.8 Å of diameter) connected by eight-membered ring (8MR) units with cage windows of 4.2–4.8 Å, in addition to four- and six-membered ring (4MR, 6MR) pore apertures (see Figure 1). Besides, the coexistence of hydrogen and chlorine atoms in the dcIM linker offers more versatile interactions with guest molecules than expected for only hydrogen bonds in ZIF-8, advancing reactivity in catalysis or selectivity for sensing applications. Yet, perhaps owning to its complex structure, this material has not been widely explored, which is surprising given that ZIF-71, due to its intrinsic hydrophobicity, provides excellent chemical stability akin to ZIF-8. While a thorough understanding of the physical properties of ZIF-8 and its underpinning lattice dynamics has been developed,^13,14^ little is known about the fundamental vibrational characteristics of ZIF-71, which is so central to understanding the physical behavior of the material, and thus there is a gap in knowledge prior to targeting specific applications.

**Figure 1:**
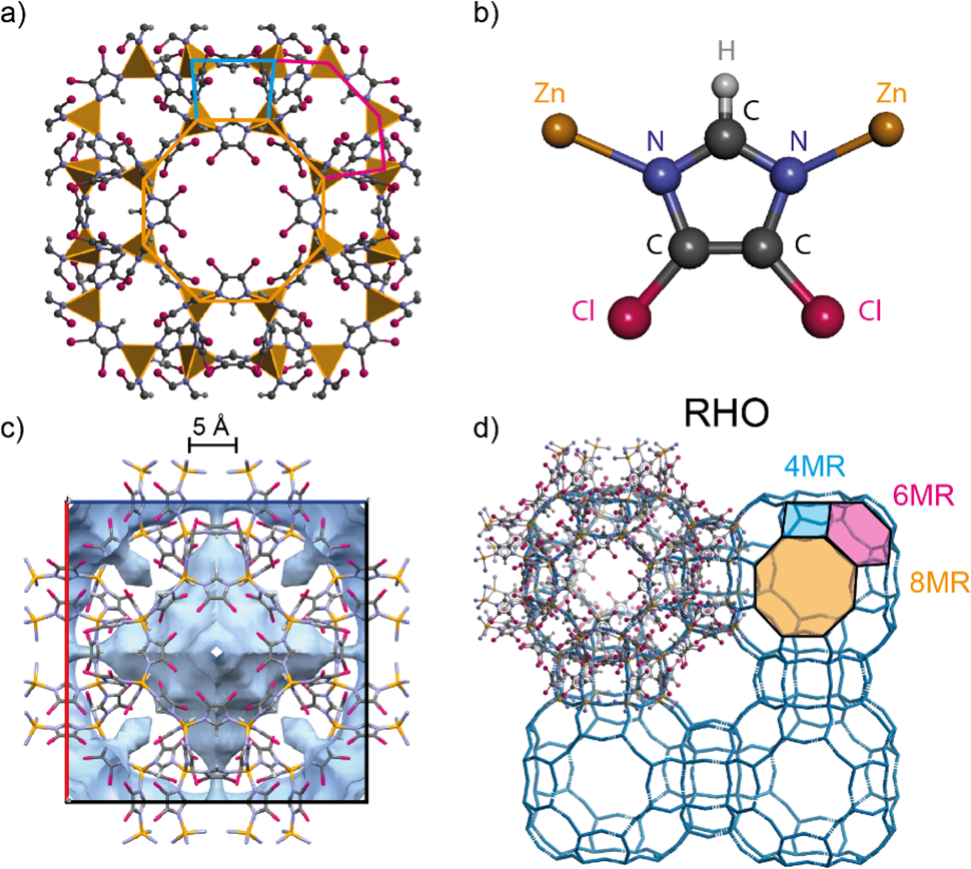
Framework structure of ZIF-71: (a) ZIF-71 unit cell where the inorganic building blocks are illustrated by the ZnN_4_ tetrahedra. (b) Building unit showing the Zn–dcIM–Zn linkages. (c) Blue surfaces denoting the nanopore, corresponding to the solvent accessible volume (SAV probe radius = 2 Å) within the open framework structure. (d) Illustration of the RHO topology, highlighting the apertures of the 4-, 6-, and 8-membered rings (MR).

In this work, we provide the first complete assignment of the vibrational modes of ZIF-71 using high-resolution neutron and synchrotron vibrational spectroscopy, in conjunction with *ab initio* quantum mechanical simulations. This multimodal approach allows us to establish the low-frequency terahertz (THz) lattice modes and unravel basic mechanistic dynamics, and further identifies the characteristic vibrational modes in the mid-infrared region. The results are important because not only they provide the missing reference for spectroscopic studies of ZIF-71, but also they hold the key to unlocking complex host–guest interactions underpinning the functions of ZIF-71.

To analyze the physical molecular vibrations corresponding to each mode of ZIF-71, we computed the theoretical spectra and vibrational frequencies using density functional theory (DFT), as implemented in a development version of the CRYSTAL17 code (running in massively parallel processing (MPP) mode on high-performance clusters).^15,16^ ZIF-71, with its 816 atoms per unit cell, is hitherto the largest MOF system for which theoretical frequency calculations with DFT have been accomplished, thereby benchmarking the CRYSTAL17 code. We have tested two all-electron basis sets of increasing size, designated as BS1 and BS2, containing 12 480 and 16 032 local functions, respectively. The calculations were performed at the B3LYP-D3 level of theory, including two- and three-body corrections (ABC) to account for dispersion interactions.^17−19^ The Fourier transform infrared spectroscopy (FTIR) data were obtained with synchrotron radiation (SR) at the MIRIAM beamline at the Diamond Light Source (Oxfordshire, U.K.). Using two different detectors (bolometer and built-in detector) and beam splitters (Mylar and KBr, respectively), the full broadband IR spectrum from 50–2000 cm^–1^ could be measured. For IR spectroscopy, the interaction between electromagnetic waves and molecular vibrations is based on dipole changes, which are only induced by asymmetric vibrations or rotations leading to the so-called selection rule. To further elucidate the symmetric modes without dipole change, or the IR inactive modes, these data sets were complemented with inelastic neutron scattering (INS) measurements, performed on the TOSCA spectrometer at ISIS Neutron & Muon Spallation Source (Oxfordshire, U.K.).^20^ Unlike optical spectroscopy techniques, all molecular motions are observed in INS without the symmetry-based selection rule; however, in practice, this technique shows dominant sensitivity to vibrations encompassing hydrogen due to the exceptionally large scattering cross-section of the hydrogen nuclei.^21^ Additionally, the vibrational dynamics in the low-energy THz region, which are so central to the structural mechanics of MOF materials, are revealed with INS, as frequencies as low as 20 cm^–1^ are measured. We further employed nanoscale analytics, such as infrared nanospectroscopy and nanoindentation, to attain the local chemical and physical information on the individual ZIF-71 crystals. Both techniques are based on atomic force microscopy (AFM), albeit operated in different modes: nanoindentation monitors the strain rates of the AFM indenter tip during the indentation process to probe the local mechanical properties, specifically the Young’s modulus (*E*) and hardness (*H*) of single crystals.^22^ Nanospectroscopy is based on a tapping-mode AFM combined with a scattering-type scanning near-field optical microscope, where the illuminated tip serves as a source for an evanescent near-field, to obtain a nanoFTIR spectrum of individual nanocrystals.^23−25^ Together, these multimodal techniques gave us a detailed “picture” of ZIF-71, comprising its intrinsic vibrational dynamics, its fundamental physicochemical behavior, and the resulting single-crystal characteristics.

As shown in Figure 2, the calculated IR spectrum yields excellent agreement with the one measured with SR-FTIR. A bulk shift of the simulated peaks to lower frequencies was applied (factor 0.98), which is a common approach considering the “nanocrystal effect”, as the strengths of real bonds, even if only slightly, are decreased from the ones of idealized crystal.^26^ For reference, the measured INS spectrum for ZIF-71 is also shown in Figure 2d. It can be seen that the shape of the predicted spectrum in the low-energy region matches remarkably well with the INS data (Figure 2c); this is a significant result given that establishing a good agreement between DFT and INS data at low wavenumbers is usually considered as a challenge even for a less complex framework.^13,27,28^ Only the combination with DFT can assign the physical motions to each observed peak, and a detailed analysis of all vibrational modes identified different characteristic spectral regions ranging from high to low energies. Above 2000 cm^–1^, a region typically associated with the stretching vibration of functional groups, C–H stretching modes are observed for ZIF-71. In the transition between functional group and the fingerprint region between 1200 and 2000 cm^–1^, the high-intensity peaks are assigned to C–N stretching modes of the aromatic ring in combination with C–H bending. Below that, the characteristic modes of the aromatic ring of dcIM are prevalent in the mid-IR fingerprint region: 900–1200 cm^–1^ for vibrations describing the in-plane ring modes and 600–900 cm^–1^ for the out-of-plane ring modes, respectively. It is further evident that the modes involving the ZnN_4_ metal clusters appear below 500 cm^–1^, where stretching and bending between Zn and N are excited at specific frequencies; here, however, the Zn atoms remain fixed, and the main resulting motions are associated with the linker units, thus slightly deforming the pores and channels of ZIF-71. Stronger structural distortions of the pores, and the framework itself, are expected in the low-energy, or THz region (<300 cm^–1^), involving the low-energy collective modes. This is precisely where relations between the physical properties and the lattice dynamics can be instigated, since soft modes (e.g., breathing modes of the framework), gate-opening, and shearing are linked with adsorption, elasticity, structural transitions, and instability.^13,28−30^ Between 170 and 280 cm^–1^, the N–Zn–N bending and stretching modes are observed introducing tetrahedral deformations, which, in turn, cause distortion of the linker unit, and some–albeit small–structural deformations of the pores will occur. Here, vibrations associated with the Cl atoms are also detected; they can play a key role for they offer additional interaction sites for guest adsorption. Stronger deformations of the 4-, 6-, and 8-membered rings (MR) are revealed in the spectral region below 150 cm^–1^ (≲ 4.5 THz): this is where intriguing physical phenomena like gate-opening, shearing, pore breathing, and other structural mechanisms underpinning the fundamental properties of the framework are prevalent.

**Figure 2:**
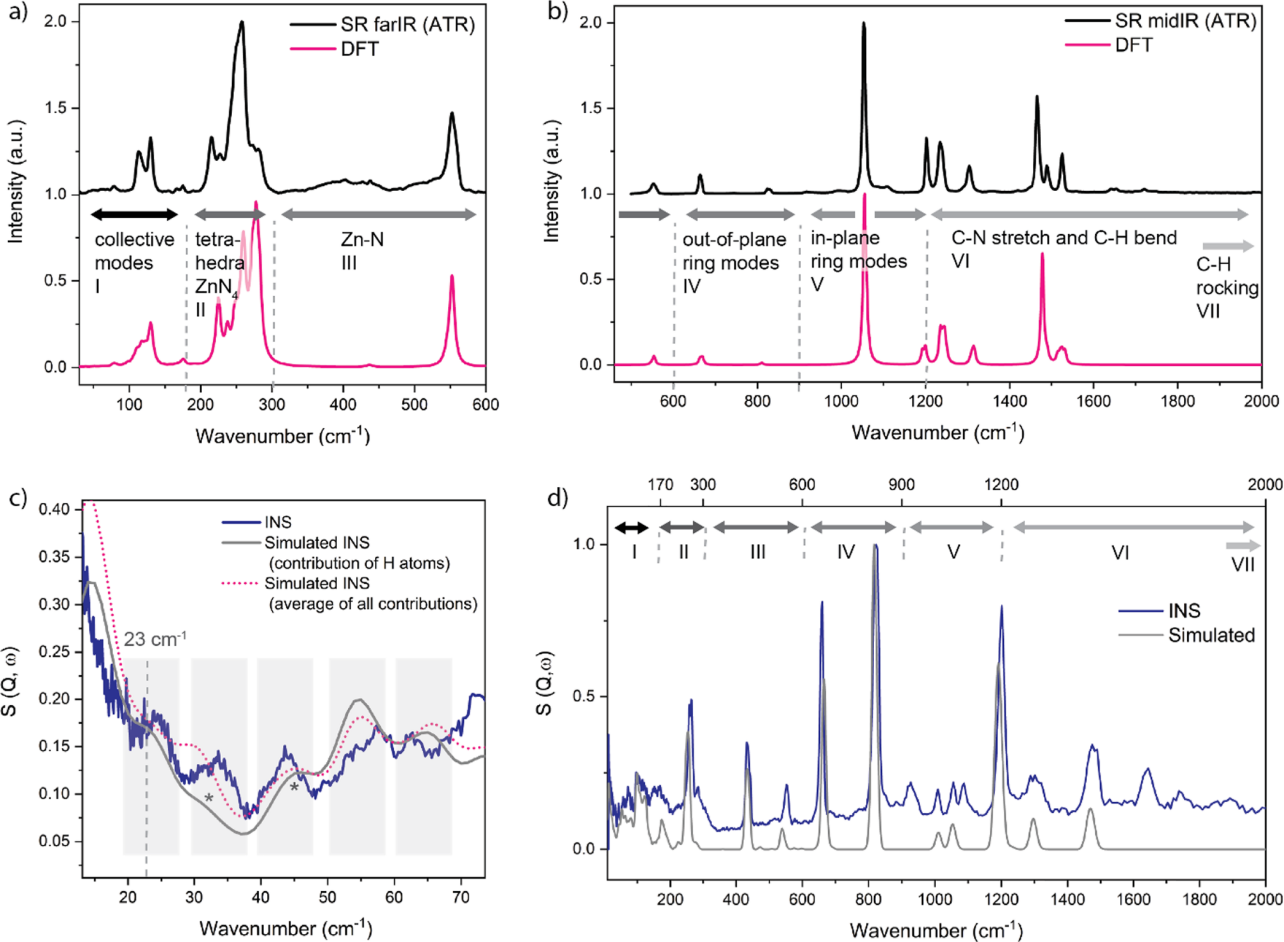
Comparison of experimental and theoretical DFT spectra for ZIF-71. (a) Far-infrared (farIR) spectrum measured with synchrotron radiation (SR) compared with DFT simulated spectrum. (b) Midinfrared (mid-IR) spectrum measured with synchrotron radiation (SR) compared with DFT simulated spectrum (shifted with a factor of 0.98). (c) Low-energy region of the spectrum obtained with inelastic neutron scattering (INS), compared with calculated INS spectra derived from the DFT phonon calculation). (d) Simulated and experimental INS spectra of ZIF-71.

Herein, we explore the low-energy collective modes of ZIF-71, which encompass contributions from the entire crystalline lattice and thus, are so intrinsically linked with the core physical phenomena observed in ZIF materials. While a full description of the vibrational modes is provided in the Supporting Information, we illustrate in Figure 3 a few crucial lattice modes that strike us as exceptional for understanding the physical phenomena of ZIF-71. Perhaps one of the most significant lattice dynamics among them is the soft mode at 10.46 cm^–1^ (∼0.3 THz), which is assigned to a strong shear deformation of the 6- and 8-membered rings (Figure 3a). Such a shearing deformation suggests a propensity to undergo a phase transformation, potentially to ZIF-72 or COK-17, which contain, in essence, the same building blocks as ZIF-71, yet their structures are entirely different: the latter manifests in a SOD topology akin to ZIF-8 but with a distorted configuration, whereas ZIF-72 is a nonporous *lcs*-type framework lacking the exceptional porosity of ZIF-71.^31,32^ Transitioning—or even structural amorphization as observed in other ZIF materials^13,33^—seems likely, especially since the shearing mode of the 8MR, which are inherently mechanically unstable subject to antiparallel shear forces given the large pore size of ZIF-71, leads to a decrease of the pore that is even more susceptible to collapse.^34,35^ As opposed to small-pore zeolites, which are characterized by eight-member ring (8MR) pores and can exhibit high thermal and mechanical stability, ZIFs are more likely to collapse to amorphization under ball-milling; a tendency that has been demonstrated for five different ZIFs.^36,37^ Our findings suggest that, in ZIF-71, too, mechanical stress can trigger shearing deformations of the 8MR leading to subsequent amorphization. This soft mode could further explain the previously observed phase transition reported during intrusion-extrusion experiments, where, despite a major collapse of the ZIF-71 framework, traces of both ZIF-71 and ZIF-72 were found by X-ray diffraction.^38^ Consistently, soft modes have been linked with structural flexibility in switchable MOFs such as DUT-8, where lattice vibrations revealed by Raman spectroscopy and computational modeling indicated differences between rigid and flexible, or porous and nonporous forms.^30^

**Figure 3:**
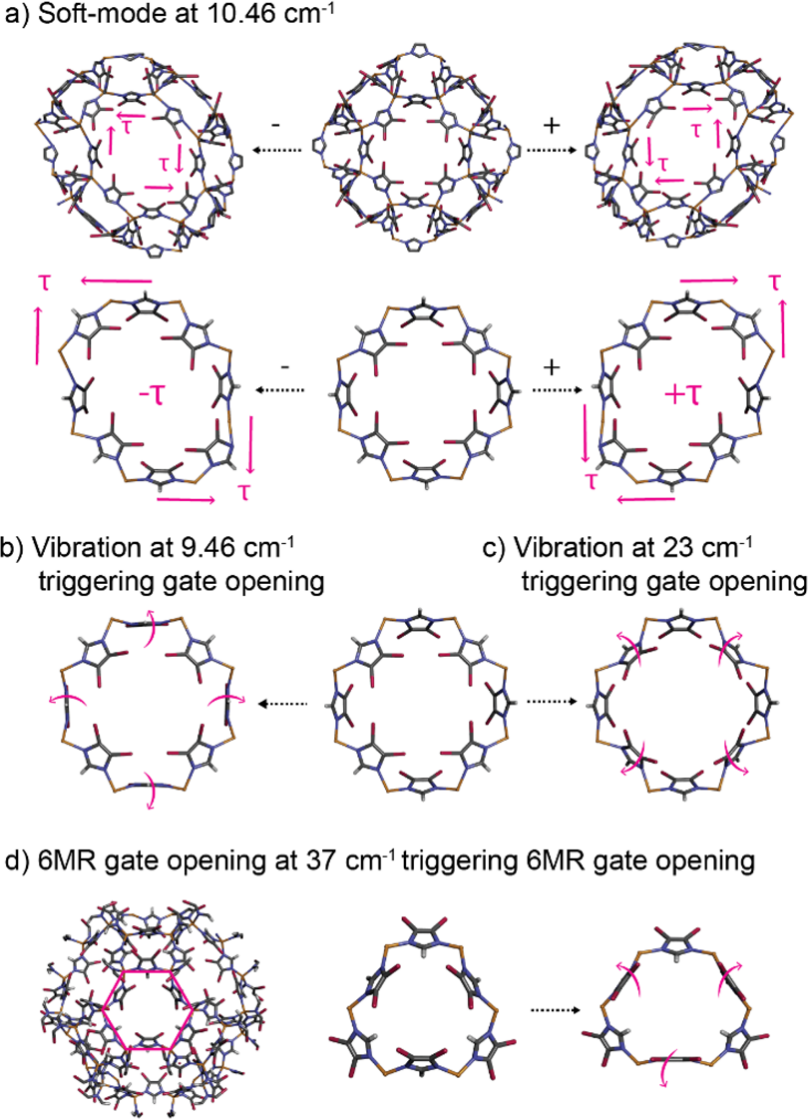
Low-energy lattice modes of ZIF-71. (a) Soft mode associated with a shear deformation of the 8MR. (b, c) Gate-opening mechanisms of the 8-membered ring (MR) via synchronous rocking of opposite organic linkers. Pink arrows designate the collective dynamics affecting the geometry of pore cavity. (d) Vibration triggering gate-opening of the 6MR through synchronous flapping of the bridging linkers.

In addition, we discovered three collective modes triggering gate-opening, a mechanism which could facilitate adsorption and significantly raise the gas uptake capacity. This is due to the synchronous flapping (or scissoring mode) of opposite ligands that results in a greater accessible pore volume via an opening of the pore aperture. For the 8MR, we detect two gate opening modes at 9.45 and 23 cm^–1^, respectively, induced due to the conformational changes of different linker units as they pivot around the metal centers (Figure 3b,c). Directly related to the increase in aperture of the 8MR, a similar, albeit less pronounced, pore breathing mechanism is propagated in the adjacent 6MR, whereas the 4MR exhibits shearing deformation. An actual gate-opening in the 6MR is however identifiable at 37 cm^–1^ (∼1 THz), where the coherent scissoring dynamics of the linker units located opposite to each other cause an increase of the pore aperture (Figure 3d). Though much less explicit, the lattice vibrations involving stretching and twisting of the Zn–N bonds and ligands also trigger pore deformations of the 4, 6 or 8MR—be it asymmetric gate-opening, pore breathing, expansion, contraction, or shearing—and while all of these mechanisms indeed distort the cage structure, they are thus far not assigned to the core physical phenomena observed in ZIF-71.

To obtain a better understanding of the physical properties of the individual ZIF-71 crystals, we performed nanoscale analytical measurements comprising near-field infrared spectroscopy and AFM nanoindentation. First, we measure, with a resolution of 20 nm, the local IR vibrational spectra: they not only reveal the homogeneity of the chemical composition of a single crystal (see the Supporting Information, Figure S7), but their average also offers comparison with conventional (far-field) ATR-FTIR techniques and the simulated spectrum, as shown in Figure 4b. For instance, the most pronounced peak at 1054 cm^–1^, assigned to in-plane ring deformation of the linker with rocking of the C–H groups, is characteristic for ZIF-71, while the smaller peaks associated with C–N stretching modes at 1201 cm^–1^ (with C–H bend), 1234 cm^–1^ (ring breathing), and 1301 cm^–1^ (stretching) are also in agreement with conventional measurements. The small discrepancies between the theoretical and experimental spectra around 1500 cm^–1^ stem from the fact that, here, symmetric stretches of the undercoordinated N–C–Cl groups at the edge of the unit cell are triggered which are not expected in the ideal, periodic crystal. Thus, the peak splitting observed in the experimental data indicates that these modes are in fact present at the crystal surface. Otherwise, the local single-crystal spectrum matches the ones measured on bulk, polycrystalline material and calculated from a periodic lattice; hence, this technique can be used for direct recognition of the vibrational modes, or the fingerprint, of ZIF-71, thereby facilitating prospective studies on the behavior of ZIF-71 with nanoscale resolution.

**Figure 4:**
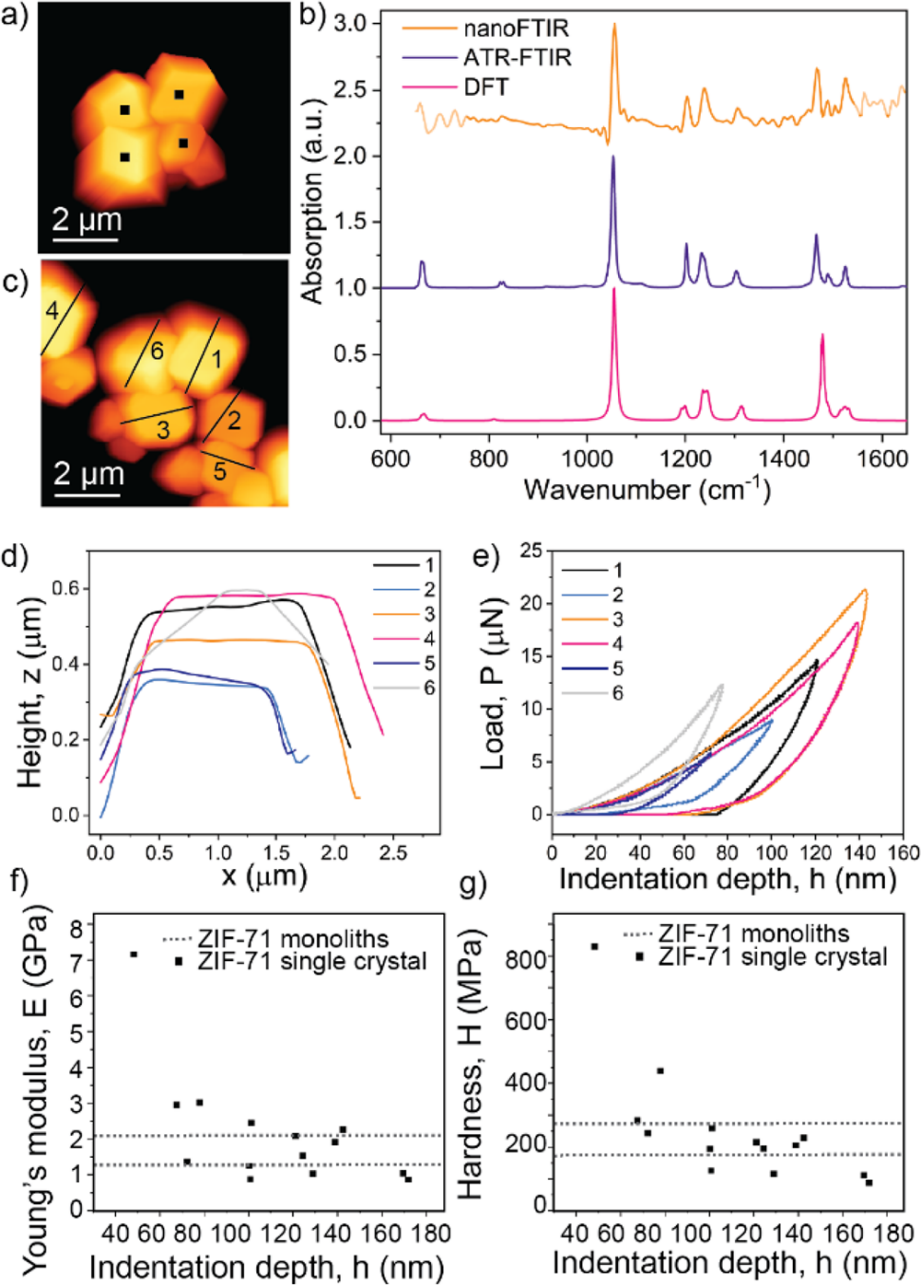
Nanoscale analytics of ZIF-71 single crystals. (a) AFM image of ZIF-71 crystals with indicated positions for nanoFTIR measurements. (b) Corresponding nanoFTIR spectra compared with ATR-FTIR measurements and DFT simulations (shifted with a factor of 0.98). (c) Individual crystals are selected for AFM nanoindentation measurements. (d) AFM height profile of the individual crystals corresponding to the lines designated in part c. (e) AFM nanoindentation load–displacement (*P*–*h*) curves of individual nanocrystals of ZIF-71. (f) Derived Young’s modulus and (g) hardness, plotted as a function of the maximum indentation depth. Dashed lines represent values measured on two different samples of ZIF-71 monoliths with instrumented nanoindentation by Tricarico et al.^39^

In addition, we measured the local mechanical properties of single ZIF-71 crystals by employing AFM nanoindentation. This technique allows us, with a resolution akin to AFM, to accurately characterize the elastic stiffness and hardness of individual ZIF-71 crystals, which is unachievable using standard techniques, since the growth of large single crystals of ZIF-71 crystals (approximately hundreds of micrometers) suitable for instrumented nanoindentation has been proven challenging up to this point. Herein, we obtain a set of load-vs-displacement (*P*–*h*) curves of several isolated ZIF-71 crystals with submicrometer size. From these shallow indentations with a surface penetration depth ranging from 60 to 170 nm, the Young’s modulus (*E*) lying in the range of of 1–3 GPa and hardness (*H*) between 100 and 300 MPa are determined using the Oliver and Pharr method, taking into account the cube-corner geometry of the diamond indenter tip.^40^ It is worth mentioning that the outlier (*E* = 8 GPa, *H* = 800 MPa) was measured on the inclined crystal (label 6 in Figure 4c–e), and introduces artifacts, as the lack of smooth and flat sample surface led to an unreliable contact area determination. Since it is unfeasible to align the small micrometer-sized crystals (through dropcasting) for orientation-specific measurement, only the exposed top surface of each crystal was probed. Owing to the difficulty to grow significantly (at least 100 times) larger ZIF-71 crystals, the mechanical properties of ZIF-71 from instrumented nanoindentation or Brillouin spectroscopy have not been reported yet, and thus, it is challenging to verify these experimental values from AFM nanoindentation. However, while this is true, it is a method that has been shown to achieve quantitative measurement on the prototypical ZIF-8, where comparison with conventional techniques and DFT calculation were feasible. With the efficacy of this method being proven, we herein report the mechanical properties of ZIF-71 with nanoscale techniques, which further substantiate these findings. Additionally, the values of Young’s modulus and hardness are in reasonable agreement with the ones measured on two different ZIF-71 monoliths (Figure 4f,g, dotted lines).^39^ While the two differing values for different samples are explained by the random orientation of nanocrystals in the monoliths and intergranular porosity, the discrepancies between monoliths and single crystals can be linked to various factors including use of different tips (Berkovich versus cube-corner geometry), nanostructure packing, anisotropic behavior of single crystals, or compliance of the AFM cantilever probe. In general, Young’s modulus of ZIF-71 (*E* ∼ 2 GPa) is notably lower than the one previously shown for ZIF-8 (3.15 GPa), an observation which we attribute to the larger pore size of ZIF-71.^14^

Knowledge of the mechanical properties paves the way for further studies targeting pressure-driven mechanical anisotropy, phase transitions, and amorphization. All of these are closely linked with THz phenomena including–but not limited to–gate-opening and shearing modes, as previously shown for ZIF-8.^35^ Additionally, the discovered gate-opening modes can finally explain the measured and computed adsorption isotherms for C_2_ (ethane, ethene) and C_3_ (propane, propene) gases in ZIF-71.^41,42^ While, at a first glance, the deviations between experiments and Monte Carlo simulations were assigned to either host–guest interactions or the complex structure of ZIF-71, there might be more to that; for C_2_ molecules, as expected, simulations predicted higher loadings, for they assume a perfect crystal structure unattainable in experiment. On the other hand, the experimental gas uptake for C_3_ molecules is higher than computationally predicted, especially if the pressure exceeds 0.3 bar. This is a strong indicator that larger molecules at higher pressure trigger bespoke gate-opening and thus, pore expansion, which ultimately leads to increased gas adsorption when compared to the simulations that assume a rigid framework. The same trend of structural flexibility can explain the simulated anomaly of adsorption of water and alcohols at varying pressure in ZIF-71.^43^ At low pressure, the affinity for water adsorption is relatively weak due to the hydrophobic, nonionic, and microporous nature of ZIF-71, where only the organic linker rather than the metal sites offer preferential adsorption sites. However, with increasing pressure, when entropy effects determine adsorption, water adsorption increases rapidly owing to its small molecular size and capillary condensation. The confinement of adsorbed molecules in a porous framework material—or, in other words, the hindrance of motion—leads to a loss of entropy;^44^ if, however, the framework is flexible upon increased pressure or temperature, entropy rises and adsorption capacity is enhanced compared to a rigid framework.

Yet, we observe less structural flexibility for ZIF-71 in comparison with the SOD-type ZIF-8, where the mIM linkers offer a higher capability to twist than the dcIM moieties. For instance, the swing angle associated with the 8MR aperture seems to be smaller when compared with the one of the 6MR of ZIF-8. In the case of ZIF-71, this could in fact facilitate the trapping of guest molecules, as a higher internal loading would not immediately lead to deformation of the pore aperture. One example is the storage or dissipation of mechanical energy, using the liquid-phase intrusion of concentrated electrolytes in a hydrophobic nanoporous framework, where the stored energy in ZIF-71 is almost doubled compared to that measured for ZIF-8.^12^ Similarly, ZIF-71 performed better than ZIF-8 in more recent impact absorbance experiments based on water intrusion: the larger water network in a ZIF-71 cage is more stabilized than in the smaller ZIF-8 cage, and accordingly, it is less favorable for a water molecule to hop to an empty neighboring ZIF-71 cage.^11^ This phenomenon increases the intrusion barrier in ZIF-71 compared to ZIF-8. Albeit less significantly, the lower flexibility in ZIF-71 could also hinder water hopping, thereby decelerating the intrusion and thus enhancing the mechanical energy absorption capacity of ZIF-71.

The above exemplar, however, is only one of many possible applications where ZIF-71 can outperform other, well-studied MOF materials and ZIF counterparts.^45^ For instance, functionalizing ZIF-8 with halogenated imidazolate linkers could increase the CO_2_ uptake, with Cl being the most stable; accordingly, in ZIF-71, the electron-withdrawing Cl groups offer adsorption sites for enhanced gas loading capacity.^46,47^ Similarly, ZIF-71 thin films, as a halogenated material, have been shown to be promising for nanofabrication of MOF devices targeting low-*k* dielectrics and photonic sensors.^48^ Our work presents the fundamental insights required prior to developing such applications and technologies by contributing a full description of the vibrational dynamics of ZIF-71. Combining DFT calculations with high-resolution synchrotron FTIR spectroscopy and inelastic neutron scattering not only completely characterizes each vibrational mode but also further unravels the key collective modes that are inherently linked with the material’s properties and functions. For instance, we discovered shearing modes with potential phase transitioning and gate-opening modes of the different cages, which could increase gas uptake. In addition, we explore the single-crystal properties of ZIF-71 using nanoscale analytical tools. This allows us, while simultaneously imaging the crystals with AFM, to locally probe the chemical composition by measuring a nanoFTIR spectrum from a 20 nm spot, and we further measured the local mechanical properties to complete the detailed picture of ZIF-71. We hope to offer the basis for—and inspire—further studies on the physical behavior of ZIF-71, as a versatile platform for basic research and application stemming from its unique topology, hydrophobicity, large pore size, and nanoscale mechanics.

## Supporting Information Available

Details about theoretical calculations and experimental methods (synthesis, PXRD, synchrotron-radiation FTIR, inelastic neutron scattering, nanoFTIR, AFM), as well as the complete assignment of vibrational modes of ZIF-71 (PDF)
Video clips of representative vibrational modes at 9.46, 10.45, 23.68, 37.42, 1076.38, and 1508.78 cm^–1^ (ZIP)
Transparent Peer Review report available (PDF)
