# Supplementary material for: Vibrational Modes and Terahertz Phenomena of the Large-Cage Zeolitic Imidazolate Framework-71
Source: J Phys Chem Lett. 2022 Mar 24;13(12):2838–44. doi: 10.1021/acs.jpclett.2c00081 (PMC9084598; doi:10.1021/acs.jpclett.2c00081)
Supplement: Supplementary file 1 — jz2c00081_si_001.pdf [file jz2c00081_si_001.pdf]

Supporting Information

for

Vibrational Modes and Terahertz Phenomena of the Large-Cage Zeolitic Imidazolate Framework-71

*Annika F. Möslein and Jin-Chong Tan**

Multifunctional Materials and Composites (MMC) Laboratory, Department of Engineering Science, University of Oxford, Parks Road, Oxford OX1 3PJ, United Kingdom

*Corresponding author: jin-chong.tan@eng.ox.ac.uk

Contents

1.1 Synthesis of ZIF-71 2

1.2 Powder X-RAY Diffraction 2

1.3 FTIR Measurements with Synchrotron Radiation 3

1.4 Inelastic Neutron Scattering Measurements 5

1.5 Density Functional Theory (DFT) Calculations 7

1.6 NanoFTIR Measurements 11

1.7 AFM Nanoindentation 12

1.8 Complete Assignment of Vibrational Modes of ZIF-71 13

1.1 SYNTHESIS OF ZIF-71

ZIF-71 was synthesised by dissolving 2.155 g of 4,5-dichloroimidazolate (dcIm) in 144.96 ml of methanol and combining it with 0.72 g of zinc acetate, likewise, diluted in 144.96 ml of methanol. The obtained white colloidal solution was stirred for 24 hours at room temperature. Nanocrystals were isolated by centrifugation at 8000 rpm for 5 minutes and washed with fresh methanol, a procedure repeated three times to remove any excess reactants. Prior to the measurements, the material was exposed to heat (80 °C) and vacuum overnight to remove any solvent.

1.2 POWDER X-RAY DIFFRACTION

The purity and crystallinity of the powdered sample were confirmed using powder X-ray diffraction (PXRD). XRD patterns were recorded using a Rigaku MiniFlex diffractometer with a Cu Kα source (1.541 Å). Measurements were performed with a step size of 0.02° and step speed of 0.01° min^{-1}.

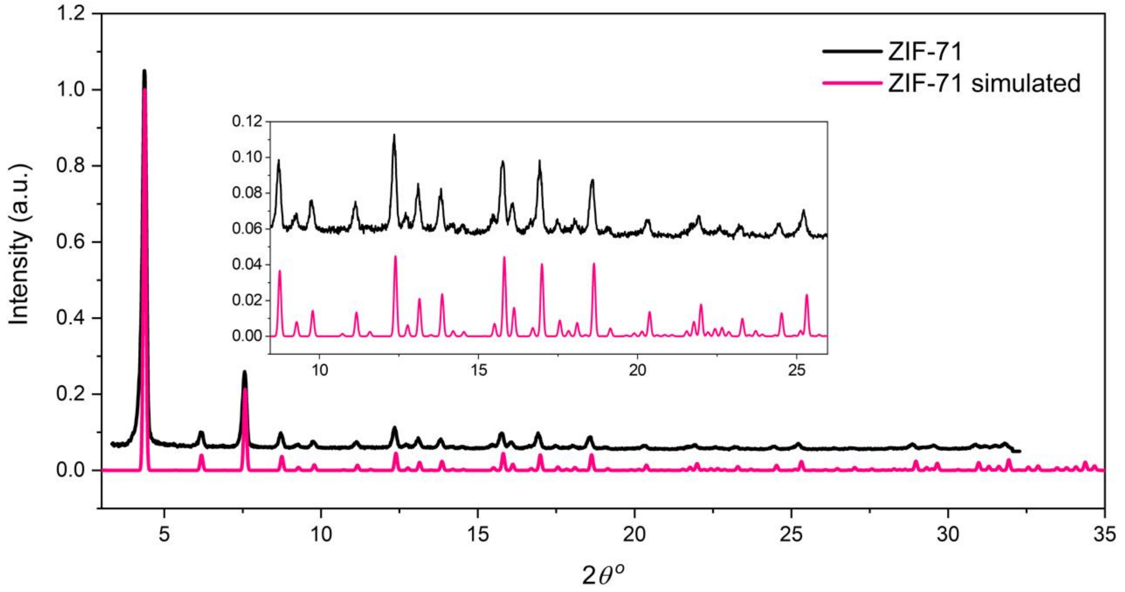

FIG S1: XRD pattern of ZIF-71.

1.3 FTIR Measurements with Synchrotron Radiation

Fourier-transform infrared (FTIR) absorption spectroscopy experiments were performed at the Multimode InfraRed Imaging and Microspectroscopy (MIRIAM) Beamline (B22) at the Diamond Light Source. The absorbance spectra were measured using a Bruker Vertex 80V Fourier Transform IR (FTIR) interferometer (Bruker Optics, Germany). For comparison, the broadband IR spectrum was also measured with specular reflectance spectroscopy using the same interferometer equipped with the Pike Technologies VeeMAX II variable angle specular reflectance accessory.[1] Absorbance spectra were measured on powder sample, and reflectance spectroscopy was probed on a pellet pressed with a (uniaxial) mechanical load of 1 ton. For both measurements, the broadband spectral range of the synchrotron source covered the visible to the low-energy THz region. A liquid helium-cooled bolometer in combination with a 6-μm Mylar multilayer beamsplitter in the interferometer allowed for measurements in the far-IR

spectral region between 0 and 650 cm^{-1}. Mid-IR measurements (550 – 4000 cm^{-1}), on the other hand, were carried out using a KBr beamsplitter and an in-built detector. IR spectra were collected with a resolution of 2 cm^{-1} and 256 scans per spectral scan. All measurements were performed at vacuum (lower than 10^{-5} bar) maintained at room temperature. Background spectra were collected by measuring a mirror before the measurements.

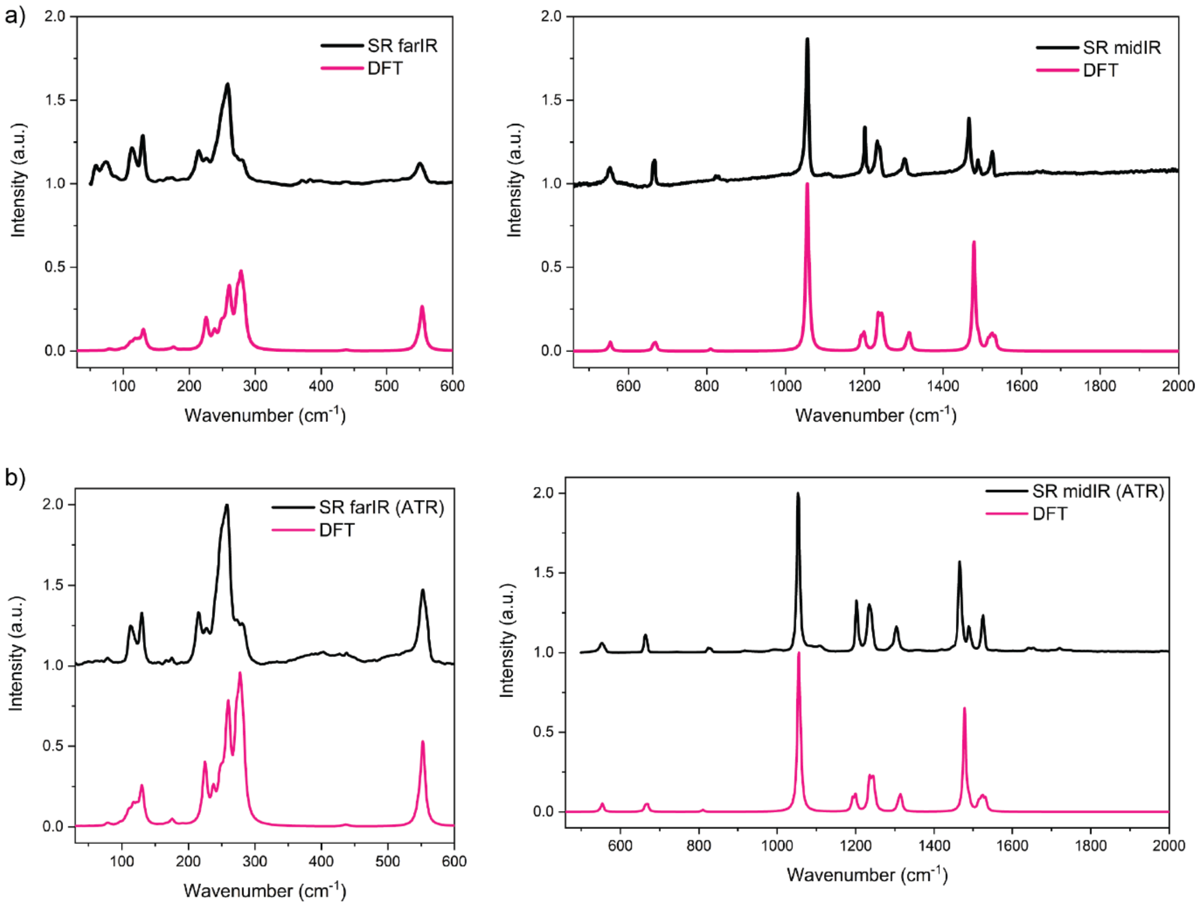

FIG S2: Comparison of synchrotron-radiation SR-IR spectroscopy: a) Imaginary part of the complex refractive index, $\kappa(\omega)$, determined from the specular reflectance spectrum *via* Kramer-Kronig Transform, b) ATR-FTIR absorbance spectrum.

1.4 Inelastic Neutron Scattering Measurements

Inelastic neuron scattering (INS) measurements were performed at the TOSCA spectrometer[2] at the ISIS Neutron and Muon Source, Rutherford Appleton Laboratory (Chilton, UK). High-resolution ($\Delta E/E$~1.25%) spectra, covering the broadband range (20-4000 cm^{-1}), were collected from the bulk polycrystalline sample (~1 g) at 10 K. The pulsed, polychromatic beam of neutrons collided with the sample and the scattered neutrons were Bragg reflected by a pyrolytic graphite analyser. Higher-order reflections beyond (002) were suppressed by a cooled ($T < 30$ K) Beryllium filter, acting as a longpass filter to analyse neutrons of a consistent final energy. Accordingly, neutrons with a final energy of ~32 cm^{-1} were passed towards the detector array composed by thirteen ^{3}He tubes with effective length of 250 mm. Five banks were located in forward direction (scattering angle ~45°) and five in backwards direction (~135°). The use of a low final energy translated into a direct relationship between energy transfer (ET, cm^{-1}) and momentum transfer (Q, $Å^{-1}$) such that ET ≈ $16Q^2$. Energy transfer and spectral intensity, i.e. $S(Q, \omega)$, were then obtained using the Mantid software.[3] The sample was wrapped in 4 cm × 4.6 cm aluminium sachet and placed into a 2.0 mm spaced flat aluminium cell, which was sealed with indium wire. To reduce the effect of the Debye-Waller factor on the experimental spectral intensity and allow comparison with the theoretical spectra, the sample cell was cooled to ~10 K by a closed cycle refrigerator. The INS spectrum was collected under vacuum over a duration of 5 hours.

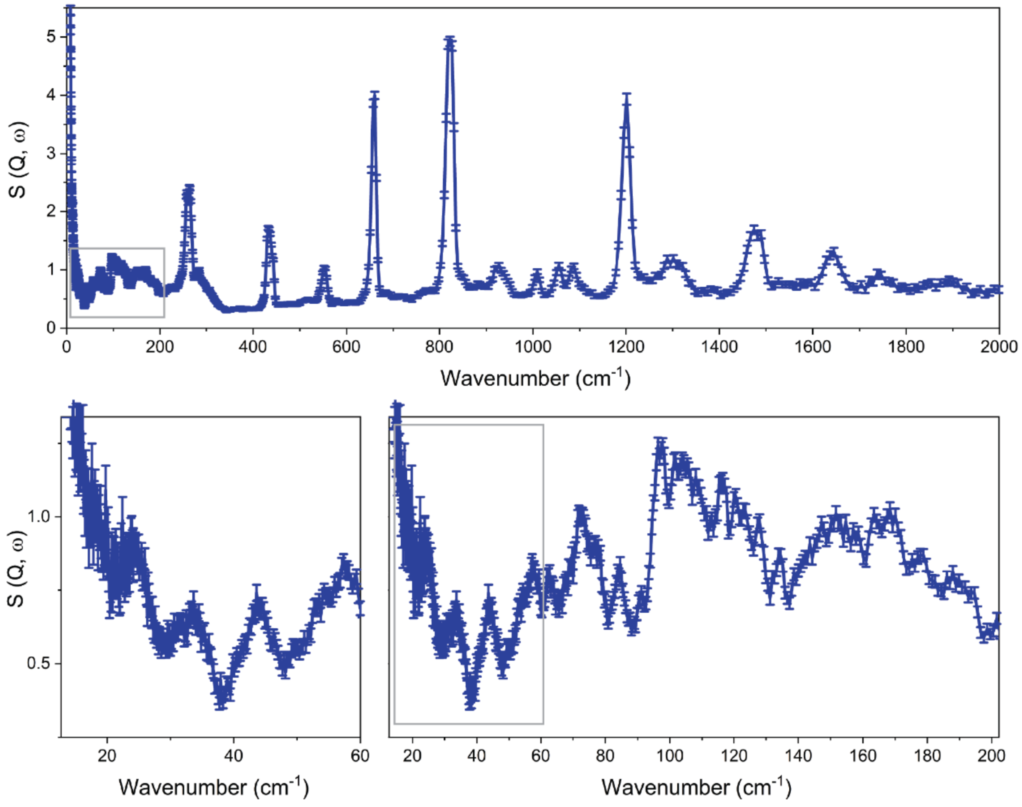

FIG S3: Inelastic neutron scattering (INS) data with error bars for a powder sample of ZIF-71.

The neutron guide upgrade of the TOSCA spectrometer, completed in 2017, has increased the neutron flux at the sample position by as much as 82 times. This upgrade improves the performance through faster measurements and by reducing the required sample mass.[4]

1.5 DENSITY FUNCTIONAL THEORY (DFT) CALCULATIONS

Ab initio density functional theory (DFT) calculations, in principle, evaluate the force-fields acting on the electrons. As the second derivative of the energy, the force constant considers the interactions emerging from electrons and nuclei within the molecule. In the DFT approach, the terms involving the Coulomb interactions between the electrons (exchange-correlation energy) are modelled by functionals employing different levels of theory. Here the crystalline orbital is approximated by a linear combination of Bloch functions, built from localized functions ("atomic orbitals"). These atomic orbitals are represented as linear combinations of Gaussian-type functions whose constant coefficients are defined by the input. Commonly used, for instance, is the B3LYP functional, a hybrid model proposed by Becke (B3)[5] to advance the gradient-corrected correlation of Lee et al. (LYP)[6]. Combined with the Grimme's dispersion correction (B3LYP-D3)[7], the DFT calculations yield theoretical IR spectra demonstrating the closest resemblance with experimental frequencies.[8] All electron basis sets were used for Zn, C, N, Cl, and H atoms, containing 12,480 (BS1) and 16,032 (BS2) local functions for the 816 atoms per unit cell.

First, the geometry optimization was carried out using a quasi-Newtonian algorithm and was considered complete when the calculation converged to the thresholds for both the RMS and maximum value for the force and atomic displacement, simultaneously. The corresponding thresholds were 3×10^{-5} (RMS on gradient), 1.2×10^{-4} (RMS on displacement), 4.5×10^{-5} (largest component of gradient), and 1.8×10^{-4} (absolute value of largest displacement). Subsequently, the IR frequencies were calculated at the special point $\Gamma = (0,0,0)$. Here, the dynamical matrix ("mass-weighted Hessian matrix") was obtained through numerically evaluating the first derivatives of the atomic gradients. The IR intensities

were then calculated with the Berry Phase approach; for a detailed description of the performed calculation, we refer to the work of Pascale et al.[9] The continuous spectrum was obtained by fitting the calculated IR intensities with Lorentzian peak shapes with a FWHM of 10 cm^{-1}. To improve the match with experimental data, the calculated IR spectra were scaled by using a scaling factor of 0.98.[10] The INS spectrum was calculated from the output of the DFT frequency calculation using the Abins v1 execution in the Mantid software.[3]

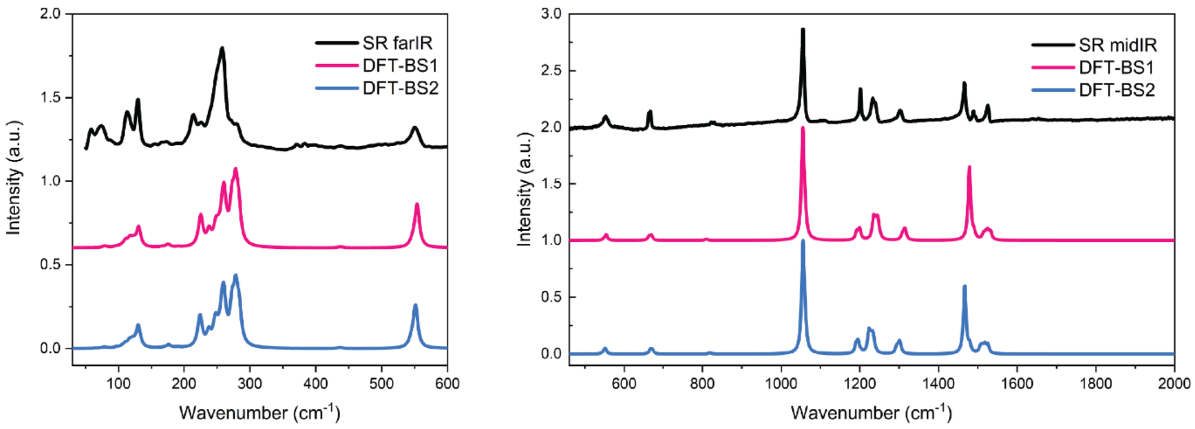

FIG S4: Comparison between infrared spectra of ZIF-71 obtained from synchrotron-radiation (SR) experiments and DFT simulations employing two different basis sets (BS1 and BS2).

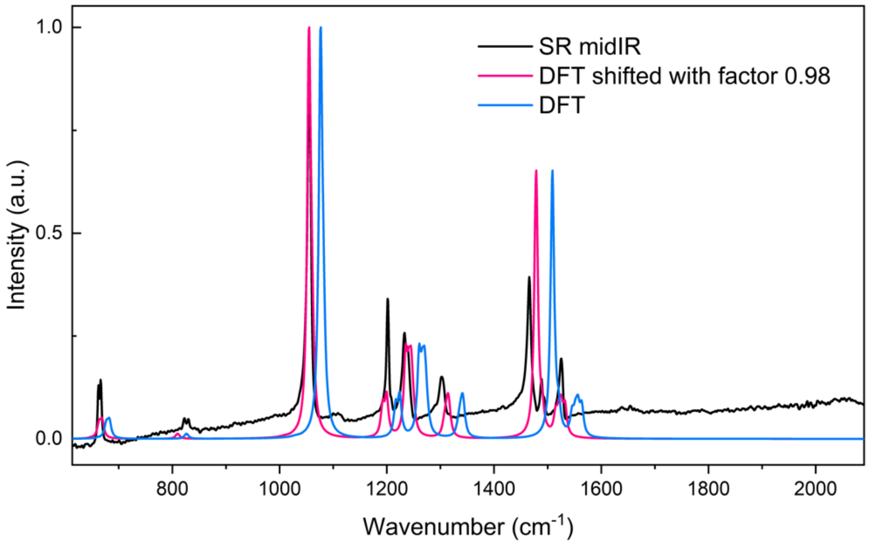

FIG S5: Bulk shift of DFT simulated spectrum versus the experimental mid-IR spectrum of ZIF-71.

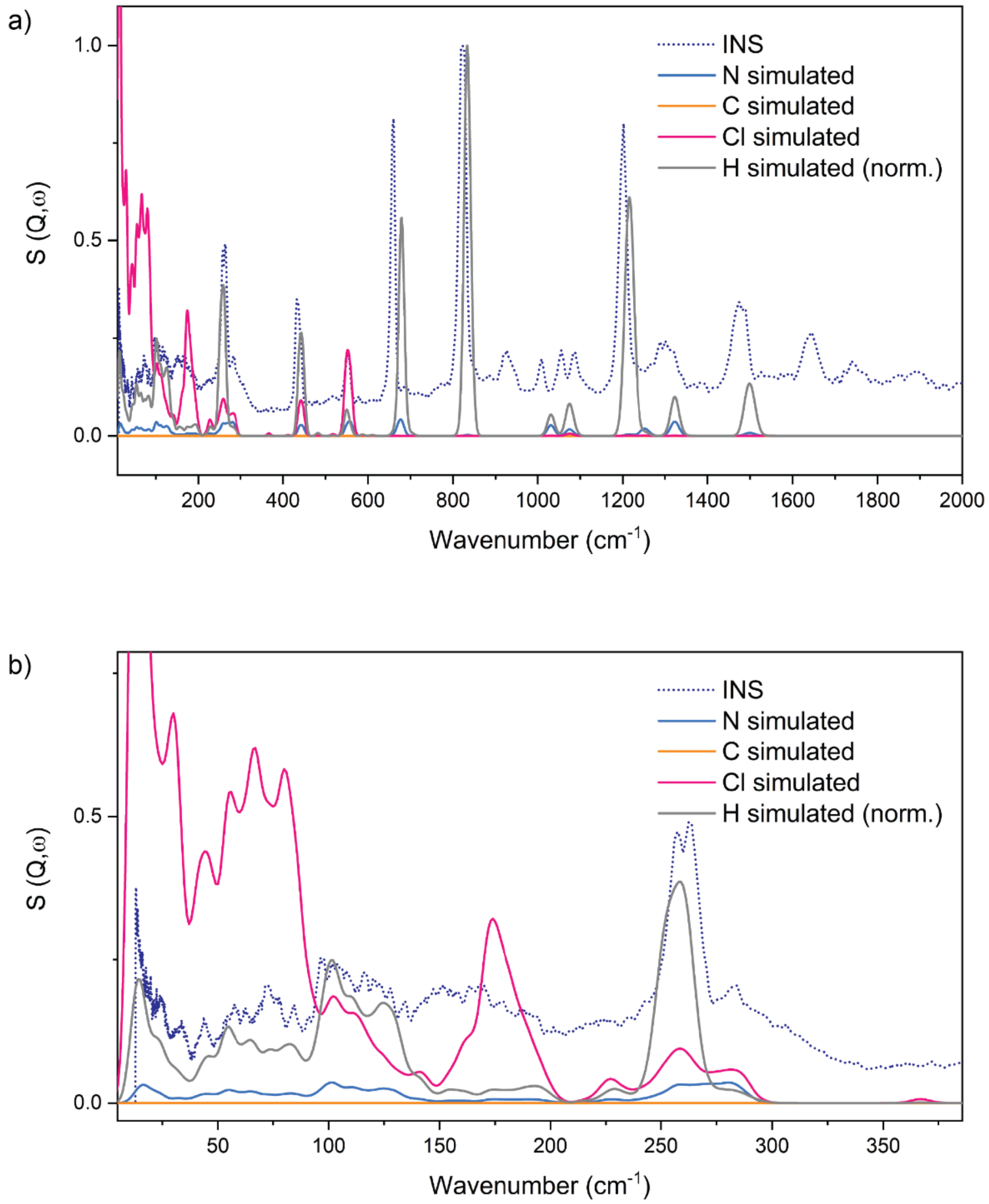

FIG S6: INS spectra derived from the phonon calculation compared with experimental measurement. No shift was applied, but the simulated spectrum for hydrogen (H) has been scaled down (normalization between 0 and 1) for better visualization.

1.6 NANOFTIR MEASUREMENTS

The near-field optical measurements were performed with a neaSNOM instrument (neaspec GmbH) based on a tapping-mode atomic force microscopy (AFM) setup where the platinum-coated tip (NanoAndMore GmbH, cantilever resonance frequency 250 kHz, nominal tip radius ~20 nm) was illuminated by a broadband femtosecond laser. The coherent mid-infrared light was generated through the nonlinear difference-frequency combination of two beams from fiber lasers (TOPTICA Photonics Inc.) in a GaSe crystal. The spectra of two laser sources covering the range from 700 to 1400 cm^{-1} and 1000 to 1600 cm^{-1}, respectively, were merged for the measurements. Demodulation of the optical signal at higher harmonics of the tip resonance frequency eliminated background contributions to yield the near-field signal, comprising amplitude and phase of the scattered wave from the tip. Employing a pseudo-heterodyne interferometric detection module, the complex optical response of the material is measured, where the real part refers to the nanoFTIR reflectance and the imaginary part depicts the nanoFTIR absorption spectrum. Each spectrum was acquired from an average of 10 Fourier-processed interferograms with 9 cm^{-1} spectral resolution, 1024 points per interferogram, and 10-ms integration time per pixel. The sample spectrum was normalized to a reference spectrum measured on the silicon substrate. All measurements were carried out under ambient conditions.

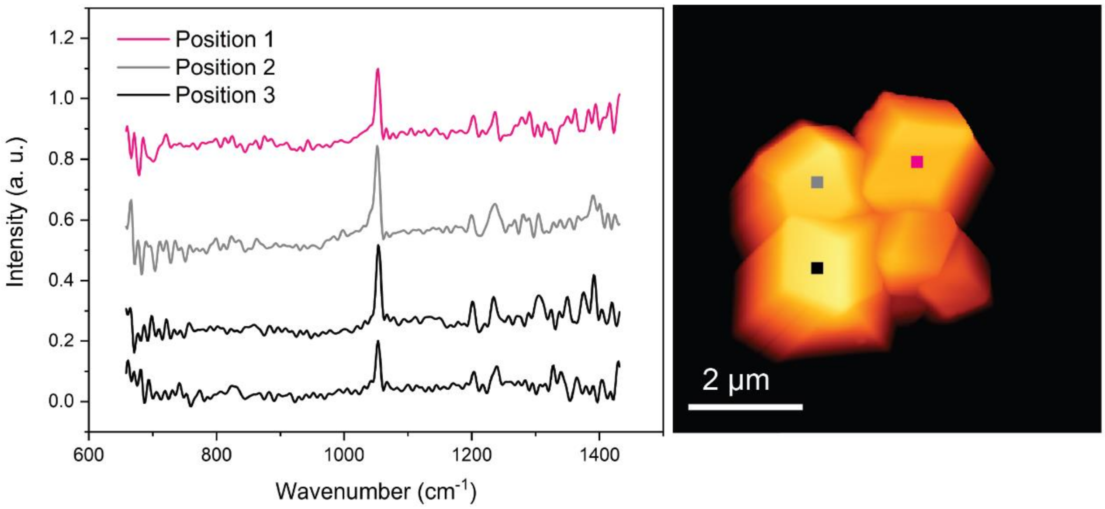

FIG S7: Local nanoFTIR spectra measured on individual ZIF-71 crystals reveal chemical homogeneity.

1.7 AFM NANOINDENTATION

AFM nanoindentation was performed with the Veeco Dimension 3100 instrument operating in indentation mode. A Bruker PDNISP probe with a cube-corner diamond indenter tip (cantilever spring constant 152 N/m, and contact sensitivity of 156.6 nm/V) was used. The nominal contact area was determined based on the indenter tip geometry established by Zeng and Tan.[11] The mechanical properties – Young's modulus (E) and hardness (H) – were derived from the set of indenter load-vs-displacement curves employing the Oliver and Pharr method, which is applicable to the cube-corner geometry.[12] For a detailed description of the AFM nanoindentation methodology for metal-organic framework crystals, we refer to the work of Zeng and Tan.[11]

1.8 COMPLETE ASSIGNMENT OF VIBRATIONAL MODES OF ZIF-71

Table S1: Complete assignment of all the vibrational modes of ZIF-71 between 0-170 cm^{-1} (<5.1 THz), 171-600 cm^{-1} (~5.1-18 THz), 601-1200 cm^{-1} (~18-36 THz), and 1201-4000 (~36-120 THz) based on the DFT simulations. Abbreviations: MR membered-ring, ν stretching, ν_s symmetric stretching, ν_a asymmetric stretching, δ bending, δ_s scissoring, ρ rocking, τ twisting, ω wagging. Simulated vibrational modes that can be observed in experimental FTIR measurements are shown in grey.

Video clips of representative vibrational modes can be found as part of the electronic SI: (in cm^{-1}) 9.46, 10.45, 23.68, 37.42, 1076.38, and 1508.78.

Collective modes 0 - 170 cm^{-1}

**Frequency** (cm^{-1})			**Intensity** (a.u.)		**Assignment**
FTIR	BS1	BS2	BS1	BS2	
	**9.46**	11.61	1	1	8MR gate-opening
	**10.45**	6.63	1	1	Soft mode with shear deformation of 8MR and 6MR
	10.52	12.7	8	6	ρ opposite linkers in 4MR, asym gate-opening
	17.25		1		ρ opposite linkers in 6MR, asym gate-opening
24	**23.68**	21.9	5	6	8MR gate-opening
	24.21	23.94	2	1	8MR, 4MR shearing, ρ Cl
	25.43	25.35	2	3	8MR breathing
33	33.06	31.95	3	5	ν_a Zn-N, N-C, ω Cl
	**37.42**	40.18	2	1	6MR gate-opening
43	40.81		3		ν_s Cl-C-N, small 6MR breathing
	46.11	47.47	2	1	ν_s Zn-N, N-C, ω Cl, small 4MR and 8MR breathing
53	51.21	53.74	5	4	flapping of neighbouring linkers, asym. gate-opening, ν_a Zn-N
	54.01	55.05	1	1	ν_a Zn-N-C, 4MR breathing
	55.68	58.95	3	1	ν_a Zn-N, 4MR and 8MR shearing, ring breathing
	61.39	62.87	2	2	ω Cl, ring breathing, 4MR and 6MR shearing
62	64.63	65.17	3	6	ring breathing, ω Cl, deformation of 8MR and 4MR
	66.35	68.83	1	3	ν_a Zn-N, ring deformation, small pore deformation
	77.37	75.83	1	5	τ Cl-C-N, ring breathing, very small pore deformation
	79.29	79.59	12	21	τ Cl, 8MR aperture increase, ν_s Zn-N: 4MR pore stretching
74	80.85	80.18	39	16	ν_a Zn-N: 4MR pore deformation
	86.14	83.13	5	3	τ Cl, ν C-H, 8MR and 4MR pore deformation, 6MR breathing
	91.56	85.82	2	1	ν_a Cl-C-N, 6MR pore deformation, 8MR and 4MR deformation
	93.31	90.73	1	1	4MR breathing, 6MR and 8MR small deformation
	99.21	98.65	34	30	ν ring, ω Cl, 4MR and 6MR aperture expansion
	103.96	102.09	4	2	6MR breathing (small)
	107.82	106.79	42	34	8MR breathing (small)
	111.95	110.15	4	7	4MR shearing, 6MR deformation
112	113.09	112.11	168	99	4MR and 6MR deformation, 8MR expansion
112	119.66	118.4	218	162	4MR and 8MR deformation, 6MR expansion
	121.91	121.28	19	19	8MR shearing, 6MR contraction
	125.35	123.79	163	171	8MR breathing, 4MR and 6MR deformation
	130.67	130.17	5	62	δ_s Cl, ν C-N, 4MR and 8MR pore stretching
129	133.15	132.41	600	651	ν Zn-N, ν N-C-H, 4MR breathing
	156.81	158.22	1	1	δ Cl, τ Zn-N, 6MR pore deformation
	161.39	163.13	10	10	ν_a Zn-N, δ_s Cl, 6MR pore deformation
	170.61	171.61	21	9	ν_a Cl, ν_a ring
	173.72	174.13	1	2	ν_a ring, δ_s Cl
	177.17	177.78	15	29	ν Zn-N δ_s Cl, 4MR and 6MR pore deformation

Vibrational modes 171 - 600 cm^{-1}

Frequency (cm^{-1})			Intensity (a.u.)		Assignment
FTIR	BS1	BS2	BS1	BS2	
	179.63	180.06	28	10	δ Zn-N (tetrahedral deformation), ring deformation
173	179.89	180.54	55	68.75	δ Zn-N bending (tetrahedral deformation), ω Cl
	182.72	183.88	2	1	v Zn-N, ring deformation
	189.79	190.2	1	7	δ Zn-N, ring stretching
190	195.41	194.86	15	17	δ Zn-N (tetrahedral deformation)
	223.49	223.06	25	20	v Zn-N, 4MR breathing
	229.01	227.38	169	272	δ Zn-N, 8MR and 6MR deformation
215	230.26	230.87	780	730	δ Zn-N, pore deformation
	236.01	234.78	2	2	δ Zn-N, $δ_s$ Cl
225	242.95	241.98	422	402	v Zn-N
247	253.13	251.87	434	597	δ Zn-N, 4MR and 8MR expansion
	254.65	253.26	77	71	δ Zn-N, ω N-C-H
	256.51	253.87	45	9	δ Zn-N, ω C-H
	256.79	254.08	5	5	δ Zn-N, ω C-H
	257.44	255.53	185	156	δ Zn-N
	262.35	260.99	138	188	ω ring, 4MR asym. gate-opening
	263.05	261.87	296	155	v_a Zn-N, ω ring
	263.63	261.87	8	140	$δ_s$ Zn-N
258	265.61	264.08	719	431	ρ linker
258	266.4	265.23	686	1082	ω linker
	269.2	268.46	87	90	v Zn-N, ring deformation
	275.26	274.57	4	2	v_a Zn-N, ring stretch
271	278.41	278.02	1165	1103	v_a Zn-N, ring and pore deformation
281	284.04	283.7	1586	1504	ρ Zn-N, ρ C-N-C, small 8MR breathing
	288.77	288.94	969	1020	τ N-C, small 6MR breathing
	439.85	438.99	3	4	ρ linker
	444.45	443.66	7	6	ρ linker
	446.1	444.83	16	17	v_a N-C-Cl, only incomplete linkers
	449.12	447.88	14	7	$δ_s$ linker
	551.7	548.38	1	1	$δ_s$ N-C, 4MR deformation
	552.08	548.59	2	10	τ N-Zn-N, 4MR expansion
	552.64	548.77	6	1	τ N-Zn-N,
	553.65	550.39	4	8	ω N-Zn-N
	554.53		1		ω N-Zn-N, ring stretching
	558.19	554.87	153	167	ω C-N, τ C-N-Zn
	560.49	557.14	14	15	v_s Zn-N (Zn fixed)
	563	559.47	120	80	v_s Zn-N (Zn fixed)
	563.53	559.85	41	95	v_s Zn-N (Zn fixed), ring shearing
	564.74	560.98	173	216	v_s Zn-N (Zn fixed), ring deformation
552	565.57	561.99	1044	1000	v_s Zn-N (Zn fixed), in-plane ring deformation

Vibrational modes 601- 1200 cm^{-1} (Ring deformations)

Frequency (cm^{-1})			Intensity (a.u.)		Assignment
FTIR	BS1	BS2	BS1	BS2	
	660.64	669.95	6	5	out-of-plane ring deformation
	661.19	670.36	19	1	out-of-plane ring deformation
	662	671.17	3	10	out-of-plane ring deformation
	663.36	672.4	5	10	out-of-plane ring deformation
	674.43	575.91	1	1	out-of-plane ring deformation
	675.12	677.05	99	8	out-of-plane ring deformation
	676.38	678.17	250	375	out-of-plane ring deformation
662	677.3	678.69	346	236	out-of-plane ring deformation
	677.77	678.96	157	354	out-of-plane ring deformation
	679.84	681.17	21	27	out-of-plane ring deformation
	680.36	681.96	65	56	out-of-plane ring deformation
666	682.82	684.58	972	761	out-of-plane ring deformation
	823.1	830.44	3	14	out-of-plane ring deformation
	824.39	831.07	58	1	out-of-plane ring deformation
821	825.09	832.29	70	116	out-of-plane ring deformation
	825.85	832.55	4	14	out-of-plane ring deformation
830	826.46	833.13	99	62	out-of-plane ring deformation
	827.29	835.16	29	81	out-of-plane ring deformation
	827.9	841.68	87	52	out-of-plane ring deformation
	1024.56	1027.14	12	8	in-plane ring deformation
	1028.59	1030.85	6	7	in-plane ring deformation
	1029.4	1031.71	4	2	in-plane ring deformation
	1029.54	1032.1	8	10	in-plane ring deformation
	1029.98	1032.58	4	5	in-plane ring deformation
	1033.14	1035.74	15	18	in-plane ring deformation
	1033.49	1063.7	3	8	in-plane ring deformation
1056	**1076.38**	1074.7	24028	24045	in-plane ring deformation, ρ H-C-N
	1080.26	1078.22	2317	2381	in-plane ring deformation, ρ H-C-N
	1081.76	1079.72	2493	2912	in-plane ring deformation, ρ H-C-N
	1084.43	1082.53	596	513	in-plane ring deformation, ρ H-C-N

Vibrational modes 1201 - 4000 cm^{-1}

	Frequency (cm^{-1})		Intensity (a.u.)		Assignment
FTIR	BS1	BS2	BS1	BS2	
1201	**1216.57**	1212.3	1839	1699	δ CH, v_a Cl-C-N, edges of unit cell
	1220.54	1216.47	1	1008	δ CH, v C-N
1201	1224.07	1218.27	2414	1666	δ CH, v C-N
	1229.18	1225.51	63	340	δ CH, v C-N
1233	**1260.24**	1245.5	3319	3431	v C-N, ring breathing
	1260.38	1245.82	1235	1174	v C-N, ring breathing
	1264.22	1249.53	46	184	v C-N
	1266.22	1251.13	2490	2191	v_a C-N
1238	1270.56	1255.91	3175	4286	v_a C-N
	1272.64	1237.63	548	411	v_a C-N
	1273.35	1257.97	368	13	v C-N
	1276.42	1260..96	486	537	v C-N
	**1332.01**	1317.33	190	1367	v C-N
	1333.06	1320.68	11	275	v_s C-N
	1334.13	1322.32	238	19	v C-N
	1334.48	1323.73	5	164	v_s C-N
1302	1335.65	1324.99	440	1741	v C-N
	1336.15	1325.64	2	500	v_s C-N
	1337.46	1325.64	78	5	v C-N
	1337.97	1326.37	300	83	v_s C-N
	1339.14	1326.95	532	371	v_s C-N
	1341.35	1328.42	1053	18	v_s C-N
	1341.77	1328.6	346	17	v C-N
1465	**1508.78**	1493.54	16845	15950	$δ_s$ N-CH
	1519.82	1505.77	22	193	$δ_s$ N-CH
1488	1520.47	1506.45	1248	1509	v C-N, δ CH
	1543.99	1532.58	74	60	v_a C-C-N
1518	1545.61	1534.23	1168	1307	v_a C-C-N, v C-C,
	1551.32	1539.22	503	468	v_s N-C-N
	1552	1539.91	630	844	v_s N-C-N
	155.49	1545.15	302	316	v C-C, v C-N,
1525	1556.6	1546.23	1331	1399	v C-C, v C-N, δ N-C-N
	1562.84	1552379	488	519	v_s N-C-Cl (edge of unit cell)
	1563.73	1553.68	1273	1423	v_s N-C-Cl (edge of unit cell)
	3277	3251.24	158	134	ρ CH
	3277.05	3251.28	15	10	v CH
	3283.44	3260.16	31	38	v CH
	3283.78	3260.53	12	14	v CH
	3296.46	3270.69	19	31	v CH
	3296.78	3270.95	2	3	v CH
	3305.46	3281.78	74	86	v CH
	3305.78	3281.96	4	3	v CH

References

(1) Babal, A. S.; Souza, B. E.; Möslein, A. F.; Gutiérrez, M.; Frogley, M. D.; Tan, J.-C. Broadband Dielectric Behavior of an Mil-100 Metal–Organic Framework as a Function of Structural Amorphization. *ACS Appl. Electron. Mater.* **2021,** *3,* 1191-1198.
(2) Parker, S. F.; Fernandez-Alonso, F.; Ramirez-Cuesta, A. J.; Tomkinson, J.; Rudic, S.; Pinna, R. S.; Gorini, G.; Fernández Castañon, J. Recent and Future Developments on Tosca at Isis. *J. Phys. Conf. Ser.* **2014,** *554.*
(3) Arnold, O.; Bilheux, J. C.; Borreguero, J. M.; Buts, A.; Campbell, S. I.; Chapon, L.; Doucet, M.; Draper, N.; Ferraz Leal, R.; Gigg, M. A.; Lynch, V. E.; Markvardsen, A.; Mikkelson, D. J.; Mikkelson, R. L.; Miller, R.; Palmen, K.; Parker, P.; Passos, G.; Perring, T. G.; Peterson, P. F.; Ren, S.; Reuter, M. A.; Savici, A. T.; Taylor, J. W.; Taylor, R. J.; Tolchenov, R.; Zhou, W.; Zikovsky, J. Mantid—Data Analysis and Visualization Package for Neutron Scattering Andμsr Experiments. *Nucl. Instrum. Methods Phys. Res.* **2014,** *764*, 156-166.
(4) Pinna, R. S.; Zanetti, M.; Rudić, S.; Parker, S. F.; Armstrong, J.; Waller, S. P.; Zacek, D.; Smith, C.; Harrison, S. M.; Gorini, G.; Fernandez-Alonso, F. The Tosca Spectrometer at Isis: The Guide Upgrade and Beyond. *J. Phys. Conf. Ser.* **2018,** *1021.*
(5) Becke, A. D. Density-Functional Exchange-Energy Approximation with Correct Asymptotic Behavior. *Phys. Rev. A, Gen. Phys.* **1988,** *38*, 3098-3100.
(6) Lee, C.; Yang, W.; Parr, R. G. Development of the Colle-Salvetti Correlation-Energy Formula into a Functional of the Electron Density. *Phys. Rev. B* **1988,** *37*, 785-789.
(7) Grimme, S.; Antony, J.; Ehrlich, S.; Krieg, H. A Consistent and Accurate Ab Initio Parametrization of Density Functional Dispersion Correction (DFT-D) for the 94 Elements H-Pu. *J. Chem. Phys.* **2010,** *132*, 154104.
(8) Matsuura, H.; Yoshida, H., Calculation of Vibrational Frequencies by Hartree–Fock-Based and Density Functional Theory. In *Handbook of Vibrational Spectroscopy*, Chalmers, J. M.; Griffith, P. R., Eds. John Wiley & Sons, Ltd: 2006; Vol. 1.
(9) Pascale, F.; Zicovich-Wilson, C. M.; Lopez, F.; Civalleri, B.; Orlando, R.; Dovesi, R. The Calculation of the Vibration Frequencies of Crystalline Compounds and Its Implementation in the CRYSTAL Code. *J. Comput. Chem.* **2004,** *25*, 888-897.
(10) Rauhut, G.; Pulay, P. Transferable Scaling Factors for Density Functional Derived Vibrational Force Fields. *J. Phys. Chem.* **1995,** *99*, 3093-3100.
(11) Zeng, Z.; Tan, J.-C. AFM Nanoindentation to Quantify Mechanical Properties of Nano- and Micron-Sized Crystals of a Metal–Organic Framework Material. *ACS Appl. Mater. Interfaces* **2017,** *9*, 39839-39854.
(12) Oliver, W. C.; Pharr, G. M. Measurement of Hardness and Elastic Modulus by Instrumented Indentation: Advances in Understanding and Refinements to Methodology. *J. Mater. Res. Technol.* **2004,** *19*, 3-20.
