# Supplementary material for: Vibrational Modes and Terahertz Phenomena of the Large-Cage Zeolitic Imidazolate Framework-71
Source: J Phys Chem Lett. 2022 Mar 24;13(12):2838–44. doi: 10.1021/acs.jpclett.2c00081 (PMC9084598; doi:10.1021/acs.jpclett.2c00081)
Supplement: Supplementary file 3 — jz2c00081_si_003.pdf [file jz2c00081_si_003.pdf]

jz-2022-00081d.R1

Name: Peer Review Information for "Vibrational Modes and Terahertz Phenomena of a Large-Cage Zeolitic Imidazolate Framework (ZIF-71)"

First Round of Reviewer Comments

Reviewer: 1

Comments to the Author

1. What is the major advance reported in the paper?

This paper describes measurement of some fundamental properties of a ZIF material, along with computer modelling. The high quality vibrational spectroscopy reported are fully assigned by a high level of computation. The authors point out that the crystal structure is the most complex MOF so far for which DFT calculations have been applied to assign vibrational frequencies.

2. What is the immediate significance of this advance?

The combined results illustrate a method that could be applied for many other hybrid materials to understand their fundamental properties.

3. Technical suggestions

The measured mechanical using nanoindentation properties come across as a separate study and are not linked to the vibrational spectroscopy or computation. The Young's moduli are presumably aggregate values since it is not clear that the crystal faces studied have been indexed, but this is not stated. The comparison with the literature is not very well explained: Figures 4f and g compare the measured values with those from study of some monoliths of the same materials, but why are there two dotted lines? Are these upper and lower bounds? I wonder also if the computation would yield elastic constants to allow calculation of Young's moduli for comparison with the measured values? This would make the manuscript more complete and make a connection with the first and last parts of the manuscript.

The English language and style of writing needs careful checking. For example:

(1) The title does not make sense to me. How do vibration modes and tetrahertz phenomena underpin a material? In what sense are these measurements 'underpinning'?

(2) The text repeatedly mentions the superior properties of ZIF-71: the hydrophobicity, large pore size, the functionality of the linkers, gas uptake, etc., but in fact none of these properties is explicitly linked

to any of the measurements made. I agree that the materials may be interesting and worthy of study but this is overplayed by the authors in what is a very good fundamental study that shows a methodology that could be used for many other materials. In fact the gate-opening effects, shearing, pore-breathing and phase transitions that would be more directly related to the conclusions made are not actually explored further. There is other related literature on ZIF-71 that could be looked at to make a link with the data presented and some real properties, such as doi 10.1002/ejic.201600695, and doi 10.1021/acs.jpcc.5b07360.

(3) Page 3 'encompasses the RHO-type cages': this is not a correct description of the structure. Figure 1 caption: 'Framework structures of ZIF-71' is not correct – there is only one structure – but rather what is shown is different representations of the structure. On the same caption it is written that the 'solvent accessible volume' is shown, but what solvent is considered?

Reviewer: 2

Comments to the Author

This paper evaluates the properties of ZIF-71, using DFT, terahertz, FT-IR and AFM to quantitatively evaluate the vibration and mechanical properties of the structure. This paper is commendable in that it provides a good description of the fundamental properties of ZIF-71, which is recognized to be highly functional among the ZIF series.

The DFT has been properly performed on a unit cell containing 816 atoms. In Figure 2, the consistency of the DFT, IR, and INS results is good, and the discussion is reliable and can contribute to a basic understanding of this material. A few points are noted below.

1. There is little discussion and explanation of the effect of the Cl group in ZIF-71. What about the effect on electron-withdrawing properties, etc.?

2. On page 10, it is explained that the 8RM of ZIF-71 is the main cause of mechanical instability. Is this argument also true for other porous materials (e.g. zeolite) that also have the 8RM? Since it should not be limited to molecular materials, it would be better to introduce other materials, discuss comparisons, and add citations.

3. At the end of the caption of Figure 4, there is a description error.

4. On page 13, there is a statement that Young's modulus of ZIF-71 is equivalent for both single crystals and monoliths. Is this similarity common in other MOFs? I would like to see a detailed explanation of this, as it relates to the interpretation of indentation results of monoliths in general.

5. The overall tone of the paper is that ZIF-71 is superior to other MOFs such as ZIF-8 in terms of capture and catalysis. However, the findings in this paper do not directly explain the discovery of these superior functions or the relationship between them. There is no need to emphasize the superiority or application over other MOFs.

Reviewer #3

Comments to author

The work by Möslein is a thorough description of THz vibrations in ZIF-71, both from a theoretical and experimental point of view.

Raman and also AFM data are supplied an give interesting insights into mechanical instabilities and flexibility.

The study is of high technical quality.

Major comments:

- Fig 3: it is misleading to label these modes as „gate opening" „6MR gate opening", because the paper discusses only elastic transformations (vibrations). But gate opening characterizes n inelastic transformation. For many scientists this is not clear when these terms are mixed. It is still very difficult and sometimes ambiguous to assign a mode (soft mode) to trigger a phase transition. This distinction should be made more clear. In the text later it is better explained „triggering gate opening"
- „8m inherently unstable", this might be overstated, as many zeolites have stable 8MR elements.

Minor comments:

- Abstract: ..“large pore size", → relatively large pore size (still small against other MOFs)
- P4: to the best of our knowledge: largest system: Not sure if that is true, is it relevant?
- Fig 2c: I am not sure if this is a „precise agreement" as mentioned in the text. There is a lot of noise. Maybe Deuterium would help.
- Fog 2a: What is the reason for the additional low frequency vibrations in the SR?
- „Dynamics can be instigated..", maybe the authors could be more precise here. „defining phenomena" what does this mean exactly. Maybe some comments on soft modes and group theory are needed here.
- „Soft mode at 10.46 cm-1" can the authors estimate the error?
- P11: In general we observe less structural flexibility → is not very precise, could be made more specific.
- P13: 3.2 GPa→ ref missing
- Some literature seems missing on collective vibrations in MOFs, Raman work etc.

Author's Response to Peer Review Comments:

DEPARTMENT OF ENGINEERING SCIENCE
UNIVERSITY OF OXFORD
PARKS ROAD
OXFORD OX1 3PJ
UNITED KINGDOM

Prof. Jin-Chong Tan
Professor of Nanoscale Engineering
Fellow of Balliol College, Oxford.
Telephone: +44 (0)1865 273925
e-mail: jin-chong.tan@eng.ox.ac.uk
URL: https://eng.ox.ac.uk/mmclab

14 February 2022

Dear Editor,

Manuscript jz-2022-00081d.R1

Thank you for the reviewers' comments and suggestions which we have now cogently addressed to improve the quality of the revised manuscript. We have added new data and additional references to further substantiate the work. The point-by-point response is summarised below.

Reviewer: 1

Recommendation: This paper is probably publishable, but major revision is needed; I do not need to see future revisions.

Comments:

1. What is the major advance reported in the paper?

This paper describes measurement of some fundamental properties of a ZIF material, along with computer modelling. The high quality vibrational spectroscopy reported are fully assigned by a high level of computation. The authors point out that the crystal structure is the most complex MOF so far for which DFT calculations have been applied to assign vibrational frequencies.

2. What is the immediate significance of this advance?

The combined results illustrate a method that could be applied for many other hybrid materials to understand their fundamental properties.

3. Technical suggestions

The measured mechanical using nanoindentation properties come across as a separate study and are not linked to the vibrational spectroscopy or computation. The Young's moduli are presumably aggregate values since it is not clear that the crystal faces studied have been indexed, but this is not stated. The comparison with the literature is not very well explained: Figures 4f and g compare the measured values with those from study of some monoliths of the same materials, but why are there two dotted lines? Are these upper and lower bounds? I wonder also if the computation would yield

elastic constants to allow calculation of Young's moduli for comparison with the measured values? This would make the manuscript more complete and make a connection with the first and last parts of the manuscript.

Response: Having acquired the vibrational spectra from the bulk, polycrystalline sample, or computationally derived from an idealised, periodic crystal, we also probed the FTIR spectrum of single crystals. Directly related with single-crystal properties, we further studied the mechanical properties of ZIF-71, which cannot be acquired with conventional techniques owing to the difficulty to grow significantly larger crystals. Therefore, we derive the Young's modulus from probing individual crystals with AFM nanoindentation. Since mounting the single crystals in specific orientations to accurately probe different facets has been proven challenging, we did not assign the values to specific facets. This piece of information has been added to the revised manuscript on page 14. In addition, we emphasise that the dotted lines in Figures 4f and g, adapted and used with permission from Tricarico et al. [39], correspond to two different samples of ZIF-71 monoliths. The discrepancies between these two samples are explained in their study by intergranular porosity and random orientation, an explanation, which, too, has been added in the manuscript on page 13. All corresponding changes in the manuscript are marked in green.

Although the studies on the molecular vibrations and single-crystal properties could potentially be separated, we strongly believe that providing a complete picture of ZIF-71 is beneficial in this manuscript. The combined knowledge of both vibrational modes and mechanical properties paves the way to in-depth investigations targeting pressure-driven mechanical anisotropy and amorphization that are so closely linked with collective modes (e.g. gate-opening and shearing instabilities), as previously shown for ZIF-8 (Maul et al. *Phys. Rev. B*, 2019, ref. 33). Unfortunately, calculating the elastic constants has been proven unfeasible for this huge MOF system to date.

The English language and style of writing needs careful checking. For example:
(1) The title does not make sense to me. How do vibration modes and tetrahertz phenomena underpin a material? In what sense are these measurements 'underpinning'?

Response: We have modified the title to 'Vibrational Modes and Terahertz Phenomena of a Large-Cage Zeolitic Imidazolate Framework (ZIF-71)'

(2) The text repeatedly mentions the superior properties of ZIF-71: the hydrophobicity, large pore size, the functionality of the linkers, gas uptake, etc., but in fact none of these properties is explicitly linked to any of the measurements made. I agree that the materials may be interesting and worthy of study but this is overplayed by the authors in what is a very good fundamental study that shows a methodology that could be used for many other materials. In fact the gate-opening effects, shearing, pore-breathing and phase transitions that would be more directly related to the conclusions made are not actually explored further. There is other related literature on ZIF-71 that could be looked at to make a link with the data presented and some real properties, such as doi 10.1002/ejic.201600695, and doi 10.1021/acs.jpcc.5b07360.

Response: Thank you for the suggestions; we have added a new paragraph focussing on how the discovered THz vibrations could be linked with previously observed adsorption anomalies in ZIF-71, based on the suggested references. We further deleted the comparison with other MOFs. The changes are marked in yellow on page 15 of the revised manuscript. We added the new references [41-44] to further explain the relevance of the THz phenomena to previous experimental observations.

(3) Page 3 'encompasses the RHO-type cages': this is not a correct description of the structure. Figure 1 caption: 'Framework structures of ZIF-71' is not correct – there is only one structure – but

rather what is shown is different representations of the structure. On the same caption it is written that the 'solvent accessible volume' is shown, but what solvent is considered?

Thank you for spotting this, we have changed the descriptions accordingly: Changes are marked in grey:

Page 3: -'encompasses the RHO-type cages': possesses a RHO-type structure

Figure 1 caption: -'Framework structure of ZIF-71'

- solvent accessible volume (SAV probe radius = 2 Å)

Additional Questions:
Urgency: High

Significance: High

Novelty: High

Scholarly Presentation: High

Is the paper likely to interest a substantial number of physical chemists, not just specialists working in the authors' area of research?: Yes

Reviewer: 2

Recommendation: This paper is probably publishable, but major revision is needed; I do not need to see future revisions.

Comments:
This paper evaluates the properties of ZIF-71, using DFT, terahertz, FT-IR and AFM to quantitatively evaluate the vibration and mechanical properties of the structure. This paper is commendable in that it provides a good description of the fundamental properties of ZIF-71, which is recognized to be highly functional among the ZIF series.
The DFT has been properly performed on a unit cell containing 816 atoms. In Figure 2, the consistency of the DFT, IR, and INS results is good, and the discussion is reliable and can contribute to a basic understanding of this material. A few points are noted below.

1. There is little discussion and explanation of the effect of the Cl group in ZIF-71. What about the effect on electron-withdrawing properties, etc.?

Response: We now emphasize the effect of the Cl groups that not only offer adsorption sites for enhanced gas uptake, but are also key to the nanofabrication of MOF devices, as shown in references [46-48]. The additional lines are marked in blue on pages 3-4 and 16.

2. On page 10, it is explained that the 8RM of ZIF-71 is the main cause of mechanical instability. Is this argument also true for other porous materials (e.g. zeolite) that also have the 8RM? Since it should not be limited to molecular materials, it would be better to introduce other materials, discuss comparisons, and add citations.

Response: We have made clear that the shearing modes can trigger amorphization in flexible ZIF materials, whereas small-cage zeolites with 8MR exhibit higher resistance to mechanical deformation (page 10). Corresponding changes are marked in green.

3. At the end of the caption of Figure 4, there is a description error.

Response: Thank you, this has been fixed (grey colour).

4. On page 13, there is a statement that Young's modulus of ZIF-71 is equivalent for both single crystals and monoliths. Is this similarity common in other MOFs? I would like to see a detailed explanation of this, as it relates to the interpretation of indentation results of monoliths in general.

Response: Since different methods are used to probe single crystals or monoliths, respectively, discrepancies can arise due to the compliance of the AFM cantilever probe, or the use of different indenter tips (Berkovich versus cube-corner geometry), which has an even larger effect on the hardness of a sample. Additional differences that stem from the nature of monoliths or single crystals can be induced by nanostructure packing in monoliths, intergranular porosity and grain boundaries, as well as the anisotropic behaviour of single crystals. Therefore, we only state that the Young's moduli are in 'reasonable agreement'. This has been emphasised on page 14, see highlighted green.

5. The overall tone of the paper is that ZIF-71 is superior to other MOFs such as ZIF-8 in terms of capture and catalysis. However, the findings in this paper do not directly explain the discovery of these superior functions or the relationship between them. There is no need to emphasize the superiority or application over other MOFs.

Response: We have deleted the comments about the superior functionality of ZIF-71, and instead, added a paragraph describing how our findings can now explain previously observed anomalies in the material's properties (in yellow on page 15).

Additional Questions:
Urgency: High

Significance: High

Novelty: Moderate

Scholarly Presentation: High

Is the paper likely to interest a substantial number of physical chemists, not just specialists working in the authors' area of research?: Yes

Reviewer #3

I think this is an excellent paper, which I would rate high. I have only minor points:

The work by Möslein is a thorough description of THz vibrations in ZIF-71, both from a theoretical and experimental point of view.
Raman and also AFM data are supplied an give interesting insights into mechanical instabilities and flexibility.
The study is of high technical quality.

Major comments:

- *Fig 3: it is misleading to label these modes as „gate opening" „6MR gate opening", because the paper discusses only elastic transformations (vibrations). But gate opening characterizes n inelastic transformation. For many scientists this is not clear when these terms are mixed. It is still very difficult and sometimes ambiguous to assign a mode (soft mode) to trigger a phase transition. This distinction should be made more clear. In the text later it is better explained „triggering gate opening"*

Response: We have changed the labels in the figure, and rephrased it in the main text. All changes in response to reviewer 3 are marked in pink, unless stated otherwise.

- *„8m inherently unstable", this might be overstated, as many zeolites have stable 8MR elements.*

Response: We made clear that this is only applicable for ZIF-71, and added a comparison with zeolites (shown in green on page 10).

Minor comments:

- *Abstract: ..“large pore size", □ relatively large pore size (still small against other MOFs)*

Response: Changed as suggested.

- *P4: to the best of our knowledge: largest system: Not sure if that is true, is it relevant?*

Response: On page 4, we have rephrased this by stating that we are benchmarking the performance of CRYSTAL17.

- *Fig 2c: I am not sure if this is a „precise agreement" as mentioned in the text. There is a lot of noise. Maybe Deuterium would help.*

Response: Page 7, we have changed it to ‘good’, also mentioning that such agreement is considered a challenge rather than stating that our match is precise.

- *Fog 2a: What is the reason for the additional low frequency vibrations in the SR?*

Response: We have added a figure in the SI (FIG S2) to compare different synchrotron-radiation IR measurements. That way, we show that the additional low frequencies stem from the techniques used rather than the sample itself. The additional changes are marked in yellow on pages S3 and S4 in the SI.

- *„Dynamics can be instigated..", maybe the authors could be more precise here. „defining phenomena" what does this mean exactly. Maybe some comments on soft modes and group theory are needed here.*

Response: We have added further details and a better explanation to page 8.

- *„Soft mode at 10.46 cm-1" can the authors estimate the error?*

Response: We already show in Table S1 in the SI that different basis sets give different wavelengths for this mode. While, at higher frequencies, the error is much smaller (1-2 cm^{-1}), the low energy collective modes below 20 cm^{-1} reveal larger deviations.

- *P11: In general we observe less structural flexibility is not very precise, could be made more specific.*

Response: We have added a comment about the different swing angles of the 8MR aperture in ZIF-71 compared with the 6MR aperture of ZIF-8 (page 15).

- *P13: 3.2 GPa ref missing*

Response: Added the omitted ref [14].

- *Some literature seems missing on collective vibrations in MOFs, Raman work etc. Inserted*

Response: We have added more details from literature on pages 8 and 10 of the revised manuscript (highlighted in pink).

Kind Regards,

J C TAN
